# Effects of l-Carnitine in Patients with Autism Spectrum Disorders: Review of Clinical Studies

**DOI:** 10.3390/molecules24234262

**Published:** 2019-11-22

**Authors:** Michele Malaguarnera, Omar Cauli

**Affiliations:** 1 Research Center “The Great Senescence”, University of Catania, 95100 Catania, Italy; Michele.Malaguarnera@gmail.com; 2 Department of Nursing, University of Valencia, 46010 Valencia, Spain; 3 Frailty and Cognitive Impairment Group (FROG), University of Valencia, 46010 Valencia, Spain

**Keywords:** neurodevelopmental disorder, vitamin, metabolism, nutritional supplementation, genetic disorders

## Abstract

Carnitine is an amino acid derivative, which plays several important roles in human physiology, in the central nervous system, and for mitochondrial metabolism, in particular. Altered carnitine metabolic routes have been associated with a subgroup of patients with autism spectrum disorders (ASD) and could add to the pathophysiology associated with these disorders. We review the current evidence about the clinical effects of carnitine administration in ASD in both non-syndromic forms and ASD associated with genetic disorders. Two randomized clinical trials and one open-label prospective trial suggest that carnitine administration could be useful for treating symptoms in non-syndromic ASD. The effect of carnitine administration in ASD associated with genetic disorders is not conclusive because of a lack of clinical trials and objectives in ASD evaluation, but beneficial effects have also been reported for other comorbid disorders, such as intellectual disability and muscular strength. Side effects observed with a dose of 200 mg/kg/day consisted of gastro-intestinal symptoms and a strong, heavy skin odor. Doses of about 50–100 mg/kg/day are generally well tolerated. Further clinical trials with the identification of the subgroup of ASD patients that would benefit from carnitine administration are warranted.

## 1. Introduction

Autism spectrum disorder (ASD) refers to a range of early-onset neurodevelopmental diseases, characterized by persistent deficits in social communication and social interaction, and restricted, repetitive patterns of behavior, interests, or activities. ASD affects approximately 1% of children and is becoming more common [1]. The causes of these disorders are still poorly understood, but growing evidence suggests that autism is a multifactorial disease influenced by genetic and environmental factors. ASD is associated with several genetic disorders [2,3], and several studies have uncovered a range of metabolic abnormalities associated with non-syndromic cases of ASD [4,5,6]. One of these, the alteration of carnitine and its derivatives, has been studied in both syndromic and non-syndromic forms of ASD [7,8,9,10,11,12]. l-carnitine is an amino acid derivative found in almost every cell in the body and it is particularly abundant in muscles, where it plays a major role in the use of fatty acid for energy production [13]. Adequate carnitine levels come from endogenous synthesis or from diet, especially meat, fish, and dairy food. Most fruits and vegetables are not rich in l-carnitine. An omnivorous diet provides about 50 to 100 mg of carnitine per day, 80% of which comes from meat, while a vegetarian diet provides around 10 mg of carnitine/day [8]. l-carnitine biosynthesis is performed with two ultimate precursors: Lysine and methionine, and the enzymatic reactions involved in this synthesis require several cofactors: vitamin C, iron, vitamin B6, and niacin [8]. l-carnitine biosynthesis involves various organelles (the nucleus, the mitochondria, the peroxisome, and the cytosol) in various tissues and organs: kidneys, liver, brain, and so forth. [14]. The biochemical role of carnitine concerns the mitochondrial metabolism of long chain fatty acids, peroxysomal beta oxidation, acetylation of histones, and protection against the deleterious effects of free radicals [15]. The first study reporting reduced serum plasma carnitine levels in 100 children with non-syndromic ASD was carried out by Filipek et al. [16], and this also demonstrated concomitant slight elevations in lactate and significant elevations in alanine and ammonia concentrations, suggesting a mild mitochondrial dysfunction. A mitochondrial defect may be the origin of the carnitine deficiency in these autistic children [11,16,17,18].

The links between l-carnitine and autism rely on three lines of evidence i.e., (1) the alteration of mitochondrial function in patients with ASD, (2) the relationships between l-carnitine/acylcarnitine levels and the symptoms of autism, and (3) the genetic aspects of autism associated with l-carnitine metabolism. These aspects have recently been reviewed in the literature [8,18,19,20,21] and will not be covered in detail in this manuscript. By contrast, the analysis of the effects of carnitine administration in ASD in both syndromic and non-syndromic forms of AD has not been reviewed. The main objectives of this review are:

(1) Evaluation of the studies in which carnitine was administered in patients with ASD (both ASD-associated with genetic causes and non-syndromic forms of ASD). We considered the protocols of administration (and co-administration with other drugs/supplements) and the patients’ characteristics.

(2) The clinical effects obtained after carnitine administration.

(3) Possible side-effects and their frequency observed after carnitine administration.

All original articles in the PubMed/Medline and Scopus electronic bibliographic databases up until September 30, 2019 were analyzed for these purposes. The following inclusion criteria were applied: (1) full text in English, (2) primary articles only, (3) identification of data regarding l-carnitine administration (supplementation) in patients with ASD. The title and abstract were analyzed to determine which articles to include. The full text was retrieved for those that fulfilled the inclusion criteria. Finally, the reference lists of all the relevant articles were manually cross-referenced to identify any additional articles. The primary search terms used were ‘carnitine’ AND ‘autis*’.

## 2. Results

### 2.1. Carnitine Administration in Non-Syndromic ASD Patients

Three clinical studies (two placebo controlled trials [22,23] and one open label trial [24]) have been published on carnitine effects in ASD with unknown genetic causes and non-syndromic forms of ASD, which are the majority of the cases of ASD (Table 1).

The first clinical trial to be published was a randomized placebo-controlled (RCT) in 2011 [22], which evaluated the effects of carnitine administration (50 mg/kg/day) or placebo for three months in children with ASD. Significant improvements in ASD symptoms were observed in professionally completed tools, such as the childhood autism rating scale (CARS), modified clinical global impression forms (CGI), and parent completed autism treatment evaluation checklist (ATEC) scores. In this study, a direct correlation between changes in serum-free carnitine levels and changes in CARS scores, cognitive function, and muscle strength was also found [22].

The second RCT evaluated the effect of higher dose of carnitine (100 mg/kg/day) or placebo in ASD patients for six months [25]. Significant improvements in autism symptoms based on the childhood autism rating scale (CARS) scores were reported but differently from the Geier et al. [22] study that showed no correlation between carnitine levels at the baseline and changes in CARS scores. Goin-Kochel et al. [24] performed an eight-week, open label trial of carnitine supplementation in male children with ASD (including one patient with a genetic cause of ASD due to TMLHE deficiency [12]) and evaluated the highest doses (200 mg/kg/day and more) on ASD symptoms after eight weeks administration. A careful and detailed psychological analysis of the participants was performed in this clinical study, including developmental/cognitive functioning, ASD symptomatology (autism diagnostic interview–revised (ADI-R) and autism diagnostic observation schedule (ADOS)), parental reported behavior over time (social communication questionnaire-current version (SCQ), the pervasive developmental disorder behavior inventory (PDDBI), and the autism impact measure (AIM), problematic behaviors were assessed using the aberrant behavior checklist (ABC). Finally, they evaluated the clinician-rated improvement/efficacy with the Clinical Global Impression Scale (CGIS, early clinical drug evaluation program version). The percentage of responders to carnitine treatment was around 60%. Most of the outcomes improved after carnitine administration in responders over time, e.g., the ABC hyperactivity subscale, SCQ, PDDBI social pragmatic problems domain, and AIM social-emotional reciprocity impact. Similarly, PDDBI social approach behaviors, PDDBI expressive social communication abilities composite scores, and PDDBI receptive/expressive social communication abilities composite scores significantly increased over time (indicating behavioral improvement). However, there were no significant changes in any scores over time after adjusting for multiple comparisons using Holm’s step-down Bonferroni correction, Nevertheless, it should be noted that a study with 10 participants makes it difficult to maintain significant effects after such an adjustment, and future trials clearly need to be performed with larger samples. Notwithstanding the above, even with such a small number of subjects (N = 10), the PDDBI learning, memory, and receptive language domain maintained a statistical significance after adjusting for multiple comparisons [22]. As regards the relationship between rises in carnitine concentration in blood after carnitine supplementation and clinical effects, there were significant correlations between increasing serum-free carnitine levels and decreasing cognitive scores (from ATEC testing) and CARS scores [22]. However, no correlation between carnitine levels at the baseline and changes in the CARS scores was reported by the other RCT, which also uses different doses [23]. In the open label trial, plasma carnitine levels were correlated with a number of PDDBI subscale scores, as well as the ABC irritability subscale [24].

### 2.2. Effect of Carnitine Administration in Genetic Disorders with ASD

Several case reports have argued that carnitine administration is beneficial for treating metabolic abnormalities in several genetic disorders with well-known causes which commonly associate ASD with other clinical manifestations [26,27,28,29,30,31,32] (Table 2).

The first relevant work on beneficial effects of carnitine administration on ASD was reported in a child with epsilon-trimethyllysine hydroxylase (TMLHE) deficiency, who also displayed episodes of neurodevelopmental regression [30]. Endogenous carnitine biosynthesis in humans proceeds through four enzymatic steps, of which the first occurs in peripheral tissue mitochondria and is catalyzed by the enzyme *N*-6-trimethyllysine dioxygenase (TMLD), which is encoded by the TMLHE gene at the Xq28 locus [34]. Mutation in TMLHE gene causes very low carnitine levels in the blood. Administration of carnitine (200 mg/kg/day) in this patient notably and gradually improved ASD symptoms (based on parents’ and clinicians’ reports) during the four-month follow-up period [30]. In the same year, Guevara-Campos et al. [26] reported on three children with mitochondrial diseases (complex II–IV) and hyperlactacidemia, who displayed ASD, hypotonia, developmental delay, and intellectual disability among other neuro-muscular symptoms, who received a cocktail of substances to improve their mitochondrial metabolism, consisting of carnitine 50 mg/Kg/day l-carnitine, a vitamin B complex, coenzyme Q10, and folic acid. An improvement in ID and hypotonia was observed over time, based on parents’ and clinicians’ reports (several years of follow-up visits), but ASD symptoms improved to a lesser extent than ID and hypotonia.

Primary carnitine deficiency (PCD) can result in a wide of spectrum of clinical manifestations ranging from early episodes with life-threatening [35] to even asymptomatic presentations [36], including reports of isolated gastrointestinal symptoms [37] and mild developmental delay [38,39]. Guevara-Campos et al. [27] recently described a case of an ASD girl with primary carnitine deficiency accompanied by intellectual disability, muscle weakness, and repeated episodes of hypoglycemia. After a diagnosis of PCD, the patient received carnitine (200 mg/kg/day) and a Vitamin B complex consisting of 50 mg each of vitamins B1 and B2, 15 mg of B3, 2 mg of B6, and 10 mg of B12), divided into two daily doses, because she did not eat fruit. After two years of follow-up, there was a noticeable improvement in muscle strength and language skills, and her IQ improved by 15 percentage points, but the patient remained inattentive and displayed a slight improvement in ASD features.

Short/branched chain acyl-CoA dehydrogenase (SBCAD) deficiency is a genetic disorder (ACADSB gene) which can manifest as symptomatic in a small subgroup of patients (approx. 10%) with neurological symptoms, including developmental delay, hypotonia, and autism, having been reported [40]. Longitudinal biochemical monitoring of the two patients with SBCAD while undergoing treatment with carnitine (100 mg/kg/day) was reported [28] but unfortunately no information on the effects of carnitine administration on ASD features and other central nervous system manifestations has yet been published.

A study by Witters et al. [31] reported that almost all patients with propionyl-CoA carboxylase deficiency (PCC gene), known as propionic aciduria, fulfilled criteria for ASD. Autistic features were present in all (N = 8) the patients with propionic aciduria, including repetitive movements or hand flapping, ritualistic behavior, stereotypical behavior (three patients), abnormal social interaction (seven patients), communication difficulties (all patients), and inflexibility in planning (all patients). Besides ASD symptoms, they display motor delay, intellectual disability, delay speech development and some of them presented hypotonia and gait impairment as the most common neurological alterations in the series. The treatment of these patients included carnitine (50–100 mg/kg/day) and biotin (10 mg/day) and a low protein diet. No information on the effects of carnitine administration on ASD features and other central nervous system manifestations was reported, but patients with early treatment and good clinical and metabolic management were less likely to develop neurological and neuropsychological features, including autistic features and ASD.

The 4q− syndrome includes interstitial and terminal deletions of the long arm of chromosome 4, and it is characterized by minor craniofacial dysmorphism and digital abnormalities, cardiac and skeletal defects, growth failure, and developmental delay, and 33% of patients had ASD [32,41,42]. Whilst no curative treatment is available, many patients are prescribed organ-specific drug therapy, and some children with 4q− syndrome receive food supplements which empirically appear to improve their well-being. These supplements include carnitine, Coenzyme Q10, omega-3 fatty acids, multivitamins [32]. The perceived benefits reported in children with 4q− syndrome consisted of increased energy levels and muscle strength and improved speech and language development. No detailed clinical evaluation was performed to objectify the effect of carnitine and other supplements.

### 2.3. Side-Effects

The analysis of RCT performed in children with non-syndromic ASD reported side-effects depending on the dose of carnitine administered. At a dose of 50–100 mg/kg/day, the patients were protocol-compliant (average adherence was >85%) [22] and generally tolerated the l-carnitine therapy well [22,23]. At a dose of carnitine 200 mg/kg/day and higher, side effects appeared [43]. One patient (10%) displayed sporadic vomiting, a strong fish odor (40–50%), and diarrhea (40–50%) and was prescribed probiotics (strains not specified) to alleviate the intestinal side effects and Fisherman’s soap (consisting essentially of soap, anise oil, mulberry juice, and cinnamon) for the unpleasant fish odor [43]. In the case of ASD-associated to genetic disorders, Ziats et al. [30] reported that doses higher than 200 mg/kg/day were not tolerated due to GI symptoms referred to as “GI discomfort” which were not related to diarrhea in the patients with TMLHE deficiency. In patients with 4q− syndrome, the side effects reported, following carnitine and other supplements administration, were mild and reversible [32].

## 3. Discussion

Vitamin or mineral supplementation is considered to be the most commonly used medical treatment for ASD, in addition to other interventions, such as pharmacological and psychological measures [44]. However, there is little evidence of a therapeutic relationship between vitamin and mineral supplementation and improvements in ASD [44,45]. This limited scientific evidence is partly related to the lack of randomized clinical trials or the analysis of relevant ASD endpoints for many of these substances. Our review suggests that carnitine administration showed some beneficial effects in core symptoms of ASD in patients with non-syndromic forms of autism [22,23,43]. The analysis of the associations between the basal carnitine level in blood/carnitine levels achieved following the treatment and behavioral changes produced mixed results [22,23,41]. These apparent discrepancies could be explained by the existence of as yet unrevealed associations between carnitine levels and some but not all ASD features [43] and/or by the fact that blood carnitine levels could not parallel those concentrations in the brain. Finally, because the amount of carnitine in the body comes from diet, it is crucial in future studies to analyze the intake of carnitine-enriched food by the study participants as a confounding variable possibly modulating the strength of these associations. The nutritional status of ASD patients has recently been considered a potential etiological factor for attention/communication disorders [6,46]. According to it, nutritional interventions, such as a ketogenic diet in ASD patients [47,48], need to evaluate carnitine intake through diet or supplemented carnitine because the high fat intake in this diet means more fatty acids need to be transported into the mitochondria for oxidation, requiring more carnitine and, therefore, increasing the risk of depletion of body carnitine stores in those ASD patients with low carnitine levels. Moreover, specific considerations, such as the features and behavior of ASD, might increase or even fall in the higher risk range, due to the sub-optimal nutritional status [44]. Interestingly, the open label trial by Goin-Kochel et al. [43] provides relevant new information about the percentage of responders to carnitine treatment, which encompasses 60% of the participants enrolled in the study and the type of symptoms mostly improved by carnitine administration. From the clinical point of view, this finding (60% of responders) pinpoints and confirms the existence of a subgroup of ASD patients with features and metabolic alterations linked to carnitine-mediated biochemical effects. One possible link to responsiveness to carnitine treatment could be alterations in the mitochondrial metabolism, which has been demonstrated in a subgroup of patients with non-syndromic ASD [4,18,49] and genetic disorders associated with ASD [20,26,50]. Most of the trials use a “mitochondrial cocktail” to improve these metabolic imbalances, and it is difficult to ascertain the substances affording the most beneficial effects until the RCTs to evaluate the effects of a compound having been performed. No generalizations on the benefits of vitamin/nutrients supplementation can be made, as, instead, a careful analysis of each substance is required. Confirming this approach, a recent systematic review on clinical trials (18 RCTs) in ASD [51] that analyzed the effect of individual vitamins on ASD reported beneficial effects for some of these compounds, but not for others. For instance, the administration of folinic acid improves ASD symptoms measured by various behavioral scales, and methyl B12 affords some improvement in ASD severity. In contrast, vitamin B6/magnesium, vitamin D3 produced inconsistent results on behavioral outcomes [46]. Several inborn errors of metabolism have been reported as increasing a patient’s risk for ASD, including lysosomal storage diseases, disorders of the creatine synthesis, disorders of purine and pyrimidine metabolism and dysfunction of the urea cycle, but autistic features are not frequently reported in organic acidemias [52,53,54]. In terms of genetic syndromes associated to ASD, data on carnitine administration has been reported for patients with TMLHE deficiency [30], with mitochondrial disorders and hyperlactacidemia [26], primary carnitine deficiency [27], propionic acidemia [31], and q4− syndrome [32]. However, carnitine was co-administered with other vitamins/cofactors, such as vitamin B groups and coenzyme Q10, in all these disorders (except in the report of the patient with TMLHE deficiency), and it is difficult to attribute the beneficial effects to carnitine alone. In contrast, the study performed on a patient with TMLHE deficiency reported significant improvements in ASD symptoms after carnitine administration. Interestingly, in the other disorders, there was improvement in ID and in muscle tone and development but ASD symptom improvement was rather limited in some reports [26,27]. Interestingly, administration of the carnitine-derivative, l-acetyl-carnitine (20–50 mg/kg/day), has been shown to improved hyperactive behavior and the improvement of social behavior in young boys with fragile X syndrome, a genetic syndrome that also include as clinical manifestation, ASD [55,56,57], and further RCTs are needed to recommend the use of l-acetylcarnitine for ASD symptoms and other psychological alterations that characterize this genetic disorder [58]. With the growing frequency of patients being diagnosed with ASD, sometimes it is difficult to exclude a coincidental diagnosis of autism in certain metabolic conditions with comorbidity, especially in cases of intellectual disability, and careful analysis is necessary when evaluating these co-morbid situations in many cases of ASD. Additional reports have confirmed the association of TMLHE mutations and decreased carnitine concentration in patients with non-syndromic ASD [59]. This finding of a metabolic aberration predisposing to ASD, with a metabolite that can be readily measured and supplemented clinically, is of significant interest for assessing the hypothesis that carnitine supplementation may improve autistic symptoms in these patients, and further RCTs are warranted.

## Figures and Tables

**Table 1:** Effects of carnitine treatment in non-syndromic ASD.

Reference	Subjects (Sex and Age)	Clinical Features	Dose and Duration of Treatment	Effect of Treatment
Geier et al., 2011 [22]	27 ASD children completed the study, (N = 16 carnitine group and N = 11 placebo group) aged from 3 to 10 years. old (23 males, 4 females).	ASD	Carnitine (50 mg/kg/day) or placebo for three months. Double-blind study.	Improvement in autism symptoms based on the childhood autism rating scale (CARS), modified clinical global impression (CGI) and autism treatment evaluation checklist (ATEC) scores.
Fahmy et al., 2013 [23,25]	30 ASD children (N = 16 carnitine group and N = 14 placebo group).	ASD	Carnitine (100 mg/kg/day) or placebo. Double-blind study. Clinical evaluation at six months.	Significant improvement in autism symptoms based on the childhood autism rating scale (CARS) scores.
Goin-Kochel et al., 2019 [24]	10 males (age range 2.7–7.7 years).	ASD (1 with TMLHE deficiency).	Start dose 200 mg/kg/day, increased up to 200 mg/kg/day (max dose 6 g/daily). Follow-up for 4 weeks and 8 weeks.	Improvements on the Clinical Global Impression Scale (CGIS) and other clinical variables.

**Table 2:** Effects of carnitine treatment on ASD associated with genetic syndrome.

Reference	Genetic Disorder	Subjects (Sex and Age)	Clinical Features	Treatment/Co-Treatment	Effect of Treatment
Ziats et al., 2015 [33]	Trimethyllysine hydroxylase epsilon (TMLHE) deficiency	1 male, 5 years (2.5–3 years when ASD diagnosis received)	ASD with episodes of neurodevelopmental regression	Carnitine 200 mg/kg/day Followed-up for 4.5 months	Notable and gradual improvements in ASD symptoms (based on parents and clinicians reports).
Guevara-Campos et al., 2015 [26]	Mitochondrial diseases (complex II–IV) and hyperlactacidemia	Three cases (two boys with 1.5 years and 1.7 years, 1 girl with 2 years)	ASD, ID, hypotonia, developmental delay, microcephaly, subtle MRI alterations.	Carnitine 50 mg/Kg/day, l-carnitine, a vitamin B complex, Coenzyme Q10, folic acid. Two patients received risperidone for their behavioral problems.	Improvements in ID and hypotonia. ASD symptoms improved less compared to ID and hypotonia. Evaluations based on parents’ and clinicians’ reports.
Witters et al., 2016 [31]	Propionic acidemia	All 8 patients had autistic features. (age range 3–21 years, 7 males and 1 female)	ASD. Motor Delay, ID, delay speech development. (some of them presented hypotonia and gait impairment)	All patients received carnitine (50–100 mg/kg/day) and biotin (10 mg/day) supplements. All the patients were on a strict protein-restricted diet.	Patients with early diagnosis, treatment, and good clinical and metabolic management will be less likely to develop neurological and neuropsychological features, including autistic features and ASD. Patients with a later diagnosis and recurrent metabolic decompensations are more frequently diagnosed with developmental delay and with ASD.
Guevara-Campos et al., 2019 [27]	Primary carnitine deficiency	1 case (girl, 7 years)	ASD, ID, muscle weakness and episodes of hypoglycemia	Carnitine (200 mg/kg/day) Vitamin B complex	Improvement in muscle weakness, language skills, and IQ improved by 15 percentage points. The patient remained inattentive and displayed a slight improvement in ASD features.
Strehle, 2011 [32]	4q-syndrome	15 children	Five out 15 children had ASD, 7 out 15 had developmental delay. Many comorbidities associated.	5 patients received Carnitine, Coenzyme Q10, omega-3 fatty acids and multivitamins	The perceived benefits of these supplements were a stronger immune system, increased energy levels and muscle strength, and improved speech and language development. No clinical evaluation performed.
